# Potential association of pulmonary tuberculosis with genetic polymorphisms of toll-like receptor 9 and interferon-gamma in a Chinese population

**DOI:** 10.1186/1471-2334-13-511

**Published:** 2013-10-31

**Authors:** Yu Yang, Xiangwei Li, Wei Cui, Ling Guan, Fei Shen, Jinsheng Xu, Feng Zhou, Mufei Li, Cong Gao, Qi Jin, Jianmin Liu, Lei Gao

**Affiliations:** 1 MOH Key Laboratory of Systems Biology of Pathogens, Institute of Pathogen Biology, Chinese Academy of Medical Sciences & Peking Union Medical College, Beijing, China; 2 Henan Provincial Hospital of Infectious diseases, Zhengzhou, China

**Keywords:** Pulmonary tuberculosis, TLR9, IFN-γ, Genetic polymorphisms, Susceptibility, Chinese

## Abstract

**Background:**

Association studies have been employed to investigate the relationships between host single nucleotide polymorphisms (SNPs) and susceptibility to pulmonary Tuberculosis (PTB). However, such candidate genetic markers have not been widely studied in Chinese population, especially with respect to the disease development from latent *M. tuberculosis* infection (LTBI).

**Methods:**

In this case–control study, 44 candidate SNPs were examined in a total of 600 participants (PTB patients, LTBI controls and healthy controls without *M. tuberculosis* infection) from Zhengzhou, China. The two groups of controls were frequency matched on gender and age with PTB patients. Genotyping was carried out by the Illumina Golden Gate assay.

**Results:**

When comparing PTB patients with LTBI controls but not healthy controls without *M. tuberculosis* infection, significant associations with disease development were observed for *TLR9* 1174 A/G, *TLR9* 1635 A/G and *IFNG* 2109G/A. The two loci in *TLR9* were in LD in our study population (r^2^=0.96, D’=1.00). A combined effect of the genotypes associated with increased risk of PTB (i.e. *TLR9* 1174G/G and *IFNG* 2109 A/A) was found when comparing PTB patients with LTBI controls (p=0.004) but not with healthy controls without infection (p=0.433).

**Conclusions:**

Potential associations between TLR9 and IFN-γ genetic polymorphisms and PTB were observed in a Chinese population which supports further study of the roles played by TLR9/IFN-γ pathway during the development of PTB.

## Background

It is estimated that one third of the world’s population is infected with *Mycobacterium tuberculosis* (*M. tuberculosis*, MTB), however, only a minority (10%) of those infected will develop tuberculosis (TB) during their lifetime [1,2]. This clinical diversity on disease susceptibility suggests that factors other than bacterial infection alone determine TB development. Apart from socio-economic factors and virulence of the pathogen, host genetic polymorphism has been thought to play an important role in determining TB susceptibility [3-5]. Association studies have been widely employed to investigate the relationships between TB and candidate genetic markers, and have been comprehensively reviewed previously [6-9]. Most such studies designed to compare genotype frequencies between TB patients and unrelated healthy controls without MTB infection [10,11]. However, it is unclear whether an association implies susceptibility for developing active disease or just acquisition of MTB infection by such study design. Actually, host genetic polymorphisms determining outcomes of infection are more important for identification of high-risk infected people susceptible to develop TB. To employ subjects with latent MTB infection (LTBI) as controls should be a reasonable way to achieve such study objectives [10,12].

In 2010, there were about 8.8 million incident cases of TB around the world, and China has the world’s second largest number of incident cases (0.9–1.2 million) (*Treatment of Tuberculosis: Guidelines for National Programmes*. World Health Organization, 2011). High-risk population identification is crucial for diseases prevention and early diagnosis. Besides to assess potential genetic markers, association study also contributes to clarify potential mechanisms underlying host defense to the disease development [13]. However, such studies addressing host genetic susceptibility to TB have not been widely conducted in China. In previous studies, several polymorphisms in the inflammatory genes, such as IL-18, TNF-α and NRAMP1 have been found to be associated with susceptibility to TB in Chinese population [14-16]. Whereas all of these studies employed healthy controls with uncertain MTB infection status and the amount of studied candidate single nucleotide polymorphisms (SNPs) were limited.

We therefore conducted the present study to assess the impacts of most widely studied SNPs, which have been studied in the other populations based on literature review, on host susceptibility to TB in Chinese. Both healthy controls without MTB infection and LTBI controls were employed to identify potential stage specific gene markers.

## Methods

### Ethic statement

This study was reviewed and approved by the Ethics Committee of the Institute of Pathogen Biology (Chinese Academy of Medical Sciences & Peking Union Medical College). Written informed consent to participate in this study and to provide 8 mL blood for research was obtained from each study participant before the interview and testing.

### Study population

In this case–control study, participants were recruited between September 2010 and February 2011 in Zhengzhou (capital city of Henan Province), China. Two hundred participants were recruited in each of the following three groups: patients with PTB, LTBI controls and healthy controls without MTB infection.

Newly diagnosed PTB patients, since September 2010, were enrolled from the outpatient tuberculosis clinic in Henna Provisional Infectious Disease Hospital (Zhengzhou). Eligibility criteria for case patients included: 1) ≥ 18 years old; 2) registered residence in Zhengzhou; 3) diagnosis of PTB based on clinical evaluation and laboratory tests (include chest X-ray, sputum smear microscopy and culture) according to the Guidelines for Implementing the Prevention and Control of Tuberculosis in China (2008, Department of Disease Control of Ministry of Health). Exclusion criteria included malignancy and diagnosis of extra-pulmonary TB cases.

Healthy controls without MTB infection and LTBI controls were registered residence in Zhengzhou and above 18 years of age. Both groups of controls were selected from participants of the general health examination and frequency matched with PTB patients on gender and 5-year age group. Healthy controls without MTB infection were tuberculin skin test (PPD test) negative (< 10 mm induration) and without evidence of active TB and history of TB. LTBI controls, without evidence of active TB and history of TB, should have a positive PPD test with diameter of the induration ≥15 mm to minimize the misclassification cased by vaccination with Bacille Calmette Guerin (BCG) [17]. Exclusion criteria for both control groups were the same as for the case group.

### Questionnaire

A questionnaire (please refer to Additional file 1), collecting information on socio-demographic characteristics, medical history, health status, family history and lifestyle factors, was completed by every participant under administration by a trained interviewer. Each study participant was assigned a unique code that was used to link the questionnaire and specimens.

### SNP selection and genotyping

The 44 target SNPs from 32 genes (see Additional file 2), which has been proposed to be associated with host susceptibility to TB in other populations, were selected based on literature review of the previous studies. To guarantee our study had 80% power to detect a positive association with an odds ratio (OR) of 2.0 (two-sided testing at α=0.05) given a sample size of 200 in each study group, it was considered that minor allele frequency of selected SNP should ≥0.15 refer to the allele frequencies in Asians from dbSNP database of the National Center for Biotechnology Information (http://www.ncbi.nlm.nih.gov/snp/).

Blood samples were collected from each participant. Genomic DNA was isolated from Buffy coat cells by QIAamp DNA Blood mini kit (QIAGEN, Milan, Italy). Selected SNPs were genotyped by the Illumina GoldenGate Genotyping Assay on an Illumina BeadXpress System. All of the assessed SNPs qualified for inclusion. Genotyping efficiency for the SNPs was between 97% and 100%. Duplicate SNPs were assessed in 27 samples and no errors were detected.

### Statistical analysis

Differences in the distribution of demographic variables and lifestyle factors between the three groups were assessed by chi-square tests. Hardy-Weinberg Equilibrium (HWE) in healthy controls without MTB infection was tested using asymptotic Pearson’s chi-square tests for each SNP. SNPs not in HWE were excluded in the data analyses. The chi-square tests were used to compare genotype frequencies of PTB patients with LTBI controls and healthy controls without MTB infection, respectively. Unconditional logistic regression analysis was used to estimate OR and corresponding 95% confidence intervals (CI) for disease development using co-dominant, dominant and recessive models for each SNP, respectively, adjusting for age and sex. In additional sensitivity analyses, the associations of the target SNPs were additionally adjusted for demographic factors which significantly related to TB in the study participants. Linkage disequilibrium (LD) was assessed by Linkage Disequilibrium Analyzer software [18]. To estimate the combined impact of the identified individual SNPs, odds of disease development were further assessed. We performed these tests on the multiplicative model, and the impact of the genotype associated with increased PTB risk was evaluated. All statistical analyses were carried out using SAS statistical software, release 9.1.

## Results

As shown in Table 1, 200 eligible patients with PTB were included in this study and the same number of healthy controls without MTB infection and LTBI controls (frequency matched on sex and 5-year age group) were enrolled. Half of the study participants (49.8%) were younger than 30 years, 55.5% of them were males. Significant differences between PTB patients and both groups of controls were observed for education (p < 0.01), per capita income in the family (p<0.01), and body mass index (p<0.01). More smokers (former smokers or current smokers) were found among PTB patients as compared with healthy controls without MTB infection (p < 0.01). As compared to PTB patients, significantly more subjects were BCG vaccinated among healthy controls without infection (p < 0.01) and LTBI controls (p < 0.01). The difference of the number of scars was marginally significant between the two groups of controls (p = 0.056).

**Table 1:** Characteristics of the study participants

**Variables**	Total (N = 600) n^*^(%)	PTB patients (N = 200) n^*^(%)	LTBI controls (N = 200) n^*^(%)	Healthy controls without MTB infection (N = 200) n^*^(%)	**p for difference**
**Age**					
18–19 years	48 (8.0)	16 (8.0)	15 (7.5)	17 (8.5)	
20–24 years	183 (30.5)	61 (30.5)	62 (31.0)	60 (30.0)	
25–29 years	68 (11.3)	23 (11.5)	23 (11.5)	22 (11.0)	
30–34 years	50 (8.3)	16 (8.0)	16 (8.0)	18 (9.0)	
35–39 years	60 (10.0)	20 (10.0)	20 (10.0)	20 (10.0)	
40–44 years	48 (8.0)	16 (8.0)	16 (8.0)	16 (8.0)	
45–49 years	65 (10.8)	22 (11.0)	22 (11.0)	21 (10.5)	
≥50 years	78 (13.0)	26 (13.0)	26 (13.0)	26 (13.0)	
**Gender**					
Male	333 (55.5)	111 (55.5)	111 (55.5)	111 (55.5)	
Female	267 (44.5)	89 (44.5)	89 (44.5)	89 (44.5)	
**Education**					
≤9 years	109 (18.2)	71 (35.5)	16 (8.0)	22 (11.0)	<0.01^§^
10–12 years	139 (23.2)	55 (27.5)	31 (15.5)	53 (26.5)	<0.01^&^
>12 years	352 (58.7)	74 (37.0)	153 (76.5)	125 (62.5)	
**Per capita income in the family**					
<500 Yuan/Month	109 (18.2)	59 (29.5)	24 (12.0)	26 (13.0)	<0.01^§^
500-2000 Yuan/Month	254 (42.3)	73 (36.5)	96 (48.0)	85 (42.5)	<0.01^&^
≥2000 Yuan/Month	237 (39.5)	68 (34.0)	80 (40.0)	89 (44.5)	
**Smoke status**					
Never smoker	429 (71.5)	131 (65.5)	146 (73.0)	152 (76.0)	0.10^§^
Former smoker	72 (12.0)	38 (19.0)	23 (11.5)	11 (5.5)	<0.01^&^
Current smoker	99 (16.4)	31 (15.5)	31 (15.5)	37 (18.5)	
**Current alcohol drinking**					
No	236 (39.4)	79 (39.5)	86 (43.2)	71 (35.5)	0.45^§^
Yes	363 (60.0)	121 (60.5)	113 (56.8)	129 (64.5)	0.41^&^
**BMI**					
≤18.5 (underweight)	78 (12.2)	42 (21.0)	17 (8.5)	14 (7.0)	<0.01^§^
18.5-25 (normal)	412 (68.7)	146 (73.0)	128 (64.0)	138 (69.0)	<0.01^&^
>25 (overweight)	115 (19.2)	12 (6.0)	55 (27.5)	48 (24.0)	
**Is there TB patient in the family**					
No	550 (91.8)	179 (89.9)	185 (92.5)	186 (93.0)	0.37^§^
Yes	49 (8.2)	20 (10.1)	15 (7.5)	14 (7.0)	0.28^&^
**Number of scars by BCG vaccination**					
0	242 (40.3)	131 (65.5)	66 (33.0)	45 (22.5)	<0.01^§^
1	309 (51.5)	60 (30.0)	117 (58.5)	132 (66.0)	<0.01^&^
≥2	49 (8.2)	9 (4.5)	17 (8.5)	23 (11.5)	

*Abbreviations*: BMI body mass index, LTBI latent M. tuberculosis infection, MTB *Mycobacterium tuberculosis*; PTB pulmonary tuberculosis, TB tuberculosis.

*Sum may not always add up to total because of missing data.

^§^p for difference between PTB patients and LTBI controls.

^&^p for difference between PTB patients and Healthy controls without MTB infeciton.

Genotyping data for all SNPs but one (rs2280788) were successfully obtained for ≥ 95% of the subjects. The minor allele frequency was < 5% for 8 assessed SNPs (rs2069778, rs4986791, rs179008, rs2066844, rs5743708, rs5030729, rs2066845, rs11574750) and genotype distribution of another 5 selected SNPs was found to be not in HWE in our study population (rs3764880, rs17235416, rs1800587, rs2234671; rs1800469). Therefore, after excluding these 14 SNPs, our following analyses were based on genotyping data of 30 SNPs as shown in Table 2 and Table 3. Genotype distribution in PTB patients was compared with LTBI controls and healthy controls without MTB infection, respectively. No significant difference in genotype distribution between the study groups was observed for any SNP.

**Table 2:** Genotype distribution of the selected SNPs in the study participants (Part 1/2)

**Selected SNP**		Genotype distribution*			**p for difference**
**SNP (ID)**	**Genotype**	**PTB patients, n (%)**	**LTBI controls n (%)**	**Healthy controls without MTB infection n (%)**	
*IL1RN*	TT	170 (85.0)	169 (85.8)	173 (88.3)	0.607^§^
9589 A/T	TA	29 (14.5)	28 (14.2)	23 (11.7)	0.432^&^
(rs454078)	AA	1 (0.5)	0	0	
*IL1B*	CC	54 (27.0)	63 (32.0)	59 (30.1)	0.270^§^
-511 C/T	CT	96 (48.0)	97 (49.2)	99 (50.5)	0.393^&^
(rs16944)	TT	50 (25.0)	37 (18.8)	38 (19.4)	
*IL2*	TT	89 (44.5)	90 (45.7)	87 (44.4)	0.972^§^
-330 T/G	TG	88 (44.0)	85 (43.2)	88 (44.9)	0.964^&^
(rs2069762)	GG	23 (11.5)	22 (11.2)	21 (10.7)	
*IL4*	TT	120 (60.0)	106 (53.8)	105 (53.6)	0.456^§^
-589 C/T	TC	70 (35.0)	79 (40.1)	77 (39.3)	0.375^&^
(rs2243250)	CC	10 (5.0)	12 (6.1)	14 (7.1)	
*IL8*	AA	74 (37.0)	75 (38.1)	68 (34.7)	0.967^§^
-251 T/A	AT	97 (48.5)	93 (47.2)	99 (50.5)	0.890^&^
(rs4073)	TT	29 (14.5)	29 (14.7)	29 (14.8)	
*IL8RB*	TT	83 (41.5)	83 (42.1)	78 (39.8)	0.985^§^
1208 C/T	TC	85 (42.5)	82 (41.6)	88 (44.9)	0.891^&^
(rs1126579)	CC	32 (16.0)	32 (16.2)	30 (15.3)	
*IL10*	TT	99 (49.5)	83 (42.1)	81 (41.3)	0.282^§^
-819 T/C	TC	80 (40.0)	94 (47.7)	87 (44.4)	0.217^&^
(rs1800871)	CC	21 (10.5)	20 (10.2)	28 (14.3)	
*IL10*	TT	176 (88.0)	162 (82.2)	160 (81.6)	0.133^§^
-1082 A/G	TC	23 (11.5)	35 (17.8)	35 (17.9)	0.201^&^
(rs1800896)	CC	1 (0.5)	0	1 (0.5)	
*IL18*	CC	157 (78.5)	147 (74.6)	152 (77.6)	0.648^§^
-137 G/C	CG	40 (20.0)	46 (23.4)	42 (21.4)	0.865^&^
(rs187238)	GG	3 (1.5)	4 (2.0)	2 (1.0)	
*IL18*	CC	58 (29.0)	50 (25.4)	49 (25.0)	0.708^§^
-607 A/C	CA	99 (49.5)	101 (51.3)	101 (51.5)	0.658^&^
(rs1946518)	AA	43 (21.5)	46 (23.4)	46 (23.5)	
*TLR1*	GG	90 (45.2)	90 (35.4)	74 (37.8)	0.325^§^
-7202 A/G	GA	84 (42.2)	91 (32.7)	103 (52.6)	0.117^&^
(rs5743551)	AA	25 (12.6)	16 (8.1)	19 (9.7)	
*TLR2*	TT	97 (48.5)	102 (51.8)	97 (49.5)	0.657^§^
597 C/T	TC	83 (41.5)	73 (37.1)	81 (41.3)	0.956^&^
(rs3804099)	CC	20 (10.0)	22 (11.2)	18 (9.2)	
*TLR5*	CC	189 (94.5)	189 (95.9)	188 (96.4)	0.502^§^
1174 C/T	CT	11 (5.5)	8 (4.1)	7 (3.6)	0.377^&^
(rs5744168)	TT	0	0	0	
*TLR9*	AA	70 (35.0)	67 (34.0)	68 (34.7)	0.051^§^
1174 A/G	AG	89 (44.5)	106 (53.8)	95 (48.5)	0.592^&^
(rs352139)	GG	41 (20.5)	24 (12.2)	33 (16.8)	
*TLR9*	GG	72 (36.0)	70 (35.5)	71 (36.2)	0.052^§^
1635 A/G	GA	88 (44.0)	104 (52.8)	93 (47.5)	0.608^&^
(rs352140)	AA	40 (20.0)	23 (11.7)	32 (16.3)	

*Abbreviations*: IL interleukin, IL1RN inteleukin 1 receptor antagonist; IL8RB interleukin 8 receptor beta; LTBI latent M. tuberculosis infection; MTB *Mycobacterium tuberculosis*, PTB pulmonary tuberculosis, SNP single nucleotide polymorphism, TB tuberculosis, TLR Toll-like receptor.

*Sum may not always add up to total because of missing data.

^§^p for difference between PTB patients and LTBI controls.

^&^p for difference between PTB patients and Healthy controls without MTB infection.

**Table 3:** Genotype distribution of the selected SNPs in the study participants (Part 2/2)

**Selected SNP**		Genotype distribution*			**p for difference**
**Gene**	**SNP (ID)**	**PTB patients, n (%)**	**LTBI controls n (%)**	**Healthy controls without MTB infection n (%)**	
*CCR2*	GG	125 (62.5)	125 (63.5)	120 (61.2)	0.397^§^
190 A/G	GA	62 (31.0)	65 (33.0)	67 (34.2)	0.612^&^
(rs1799864)	AA	13 (6.5)	7 (3.6)	9 (4.6)	
*CCR5*	GG	71 (35.5)	62 (31.5)	59 (30.1)	0.511^§^
59029 A/G	GA	97 (48.5)	107 (54.3)	102 (52.0)	0.515^&^
(rs1799987)	AA	32 (16.0)	28 (14.2)	35 (17.9)	
*RANTES*	GG	77 (38.5)	63 (32.0)	74 (37.8)	0.349^§^
-403 G/A	GA	96 (48.0)	101 (51.3)	95 (48.5)	0.988^&^
(rs2107538)	AA	27 (13.5)	33 (16.8)	27 (13.8)	
*MCP1*	GG	71 (35.5)	64 (32.5)	75 (38.3)	0.632^§^
2518 A/G	GA	96 (48.0)	104 (52.8)	88 (44.9)	0.812^&^
(rs1024611)	AA	33 (16.5)	29 (14.7)	33 (16.8)	
*NRAMP1*	GG	151 (75.5)	151 (76.7)	139 (70.9)	0.837^§^
D543N	GA	47 (23.5)	43 (21.8)	55 (28.1)	0.582^&^
(rs17235409)	AA	2 (1.0)	3 (1.5)	2 (1.0)	
*P2X7*	AA	116 (58.0)	113 (57.4)	109 (55.6)	0.502^§^
1513 A/C	AC	76 (38.0)	71 (36.0)	76 (38.8)	0.722^&^
(rs3751143)	CC	8 (4.0)	13 (6.6)	11 (5.6)	
*P2X7*	CC	92 (46.2)	86 (43.7)	86 (43.9)	0.835^§^
-762 T/C	CT	81 (40.7)	86 (43.7)	88 (44.9)	0.669^&^
(rs2393799)	TT	26 (13.1)	25 (12.7)	22 (11.2)	
*TNF-α*	GG	169 (84.5)	178 (90.4)	176 (89.8)	0.210^§^
-308 G/A	GA	29 (14.5)	18 (9.1)	19 (9.7)	0.284^&^
(rs1800629)	AA	=2 (1.0)	1 (0.5)	1 (0.5)	
*IFNG*	AA	74 (37.0)	57 (28.9)	73 (37.2)	0.119^§^
2109 G/A	AG	97 (48.5)	99 (50.3)	85 (43.4)	0.374^&^
(rs1861494)	GG	29 (14.5)	41 (20.8)	38 (19.4)	
*CD4*	CC	188 (94.0)	185 (93.9)	184 (93.9)	0.593^§^
868 C/T	CT	11 (5.5)	12 (6.1)	12 (6.1)	0.593^&^
(rs28919570)	TT	1 (0.5)	0	0	
*CD14*	TT	54 (27.1)	66 (33.5)	60 (30.6)	0.331^§^
159 C/T	TC	103 (51.7)	97 (49.2)	104 (53.1)	0.438^&^
(rs2569190)	CC	42 (21.1)	34 (17.3)	32 (16.3)	
*MBL*	CC	150 (75.0)	150 (76.1)	141 (71.9)	0.964^§^
-221 G/C	CG	48 (24.0)	45 (22.8)	52 (26.5)	0.742^&^
(rs7096206)	GG	2 (1.0)	2 (1.0)	3 (1.5)	
*TIRAP*	CC	196 (98.0)	190 (96.5)	188 (96.4)	0.346^§^
539 C/T	CT	4 (2.0)	7 (3.6)	7 (3.6)	0.359^&^
(rs8177374)	TT	0	0	0	
*NFKBIA*	AA	150 (75.0)	148 (75.1)	153 (78.1)	0.999^§^
-881 A/G	AC	48 (24.0)	47 (23.8)	43 (21.9)	0.322^&^
(rs3138053)	CC	2 (1.0)	2 (1.0)	0	
*NFKBIA*	CC	149 (74.9)	148 (75.1)	153 (78.1)	0.998^§^
-826 C/T	CT	48 (24.1)	47 (23.8)	43 (21.9)	0.316^&^
(rs2233406)	TT	2 (1.0)	2 (1.0)	0	

*Abbreviations*: CD cluster of differentiation, CCR chemokine (C-C motif) receptor, IFKBIA NF-kappa-B inhibitor alpha, IFNG Interferon-gamma, MBL mannose binding lectin, MCP monocyte chemotactic protein, NRAMP natural resistance-associated macrophage protein, LTBI latent M. tuberculosis infection, MTB *Mycobacterium tuberculosis*, PTB pulmonary tuberculosis, RANTES regulated upon activation, normal T-cell expressed, and secreted, SNP single nucleotide polymorphism, TB tuberculosis, TLR Toll-like receptor, TNF Tumor necrosis factor.

*Sum may not always add up to total because of missing data.

^§^p for difference between PTB patients and LTBI controls.

^&^p for difference between PTB patients and Healthy controls without MTB infection.

Associations between each SNPs and the development of PTB were assessed in co-dominant, dominant and recessive models after control for age and gender. When comparing PTB patients with LTBI controls but not healthy controls without MTB infection, significant associations with developing disease were observed for *TLR9* 1174 A/G, *TLR9* 1635 A/G and *IFNG* 2109 G/A. As shown in Table 4, a significant association was observed for *TLR9* 1174 A/G with an adjusted OR of 1.87 (95% CI: 1.08–3.23) (p = 0.027) for G allele carriers when compared with the more frequent AA genotype carriers. A significantly increased risk of PTB development was observed for *TLR9* 1635 AA genotypes carrying cases compared with G allele carriers, with an adjusted OR of 1.90 (95% CI: 1.09-3.31) (p = 0.025). These two loci in *TLR9* were in LD in our study population (r^2^ = 0.96, D′ = 1.00). In addition, the GG genotype of *IFNG* 2109 G/A was found to be associated with decreased risk of disease development with an adjusted OR of 0.54 (95% CI: 0.30-0.98) (p = 0.042) when compared with AA genotype. After additional adjustment for demographic factors which significantly related with the development of TB as shown in Table 1 (i.e. education level, income, body mass index, smoking and the number of BCG vaccination scars), the associations of the SNPs were not substantially changed. In the sensitivity analyses, the findings were not changed as well when not additionally controlling for smoking which was found not to be significantly different between PTB patients and healthy controls without MTB infection (data not shown). None of the other SNPs was found to be significantly related to PTB with respect to LTBI controls or healthy controls without MTB infection. Due to the space limitation, please refer to Additional file 3 for the results of association analyses on the rest SNPs.

**Table 4:** **The association of *TLR9 *** **and *IFNG *** **SNPs with the development of pulmonary tuberculosis**

**Genotype**		**PTB patients, n (%)**	**LTBI controls n (%)**	Adjusted OR* (95% CI) p value	**Adjusted OR**^**&**^**(95% CI) p value**	**PTB patients n (%)**	**Healthy controls without MTB infection n (%)**	Adjusted OR* (95% CI) p value	**Adjusted OR**^**&**^**(95% CI) p value**
*TLR9* 1174 A/G (rs352139)	AA	70 (35.0)	67 (34.0)	Ref.	Ref.	70 (35.0)	68 (34.7)	Ref.	Ref.
	AG	89 (44.5)	106 (53.8)	0.80 (0.52-1.25)p = 0.325	0.74 (0.44-1.25) p = 0.264	89 (44.5)	95 (48.5)	0.91 (0.58-1.41) p = 0.668	0.67 (0.39-1.15) p = 0.149
	GG	41 (20.5)	24 (12.2)	1.64 (0.19-3.01) p = 0.110	1.72 (0.81-3.64) p = 0.155	41 (20.5)	33 (16.8)	1.21 (0.69-2.13) p = 0.511	0.98 (0.49-1.96) p = 0.953
	Dominant	GG + AG vs AA		0.96 (0.63-1.45) p = 0.833	0.90 (0.55-1.48) p = 0.687			0.99 (0.65-1.49) p = 0.949	0.75 (0.45-1.24) p = 0.263
	Recessive	GG vs AG + AA		**1.87 (1.08-3.23) p = 0.027**	**2.04 (1.02-4.06) p = 0.043**			1.28 (0.77-2.13) p = 0.346	1.22 (0.65-2.27) p = 0.536
*TLR9* 1635 A/G (rs352140)	GG	72 (36.0)	70 (35.5)	Ref.	Ref.	72 (36.0)	71 (36.2)	Ref.	Ref.
	GA	88 (44.0)	104 (52.8)	0.82 (0.53-1.27) p = 0.377	0.80 (0.47-1.34) p = 0.391	88 (44.0)	93 (37.5)	0.93 (0.60-1.45) p = 0.749	0.72 (0.42-1.23) p = 0.223
	AA	40 (20.0)	23 (11.7)	1.70 (0.92-3.12) p = 0.091	1.74 (0.82-3.69) p = 0.152	40 (20.0)	32 (16.3)	1.24 (0.70-2.18) p = 0.468	1.00 (0.50-2.00) p = 1.00
	Dominant	AA + GA vs GG		0.98 (0.65-1.48) p = 0.920	0.95 (0.58-1.56) p = 0.839			1.01 (0.67-1.52) p = 0.964	0.79 (0.48-1.30) p = 0.353
	Recessive	AA vs GA + GG		**1.90 (1.09-3.31) p = 0.025**	1.97 (0.98-3.96) p = 0.057			1.29 (0.77-2.15) p = 0.341	1.20 (0.64-2.45) p = 0.579
*IFNG* 2109 G/A (rs1861494)	AA	74 (37.0)	57 (28.9)	Ref.	Ref.	74 (37.0)	73 (37.2)	Ref.	Ref.
	AG	97 (48.5)	99 (50.3)	0.75 (0.48-1.18) p = 0.212	0.57 (0.33-0.99) p = 0.045	97 (48.5)	85 (43.4)	1.13 (0.73-1.74) p = 0.591	1.22 (0.71-2.07) p = 0.470
	GG	29 (14.5)	41 (20.8)	**0.54 (0.30-0.98) p = 0.042**	**0.44 (0.21-0.91) p = 0.027**	29 (14.5)	38 (19.4)	0.75 (0.42-1.34) p = 0.334	0.74 (0.37-1.51) p = 0.413
	Dominant	GG + AG vs AA		0.69 (0.45-1.06) p = 0.087	**0.53 (0.32-0.90) p = 0.018**			1.01 (0.67-1.52) p = 0.960	1.06 (0.65-1.74) p = 0.814
	Recessive	GG vs AG + AA		0.65 (0.38-1.09) p = 0.100	0.62 (0.33-1.18) p = 0.145			0.70 (0.41-1.20) p = 0.194	0.67 (0.35-1.28) p = 0.228

*Abbreviations*: CI confidence interval, LTBI latent M. tuberculosis infection, MTB *Mycobacterium tuberculosis*, OR odds ratio, PTB pulmonary tuberculosis, SNP single nucleotide polymorphism, TB tuberculosis.

*Partly adjusted for age and gender.

^&^Fully adjusted for age, gender, education level, income, body mass index, number of BCG vaccination scars and smoking.

The potential combined effect of *TLR9* 1174 A/G and *IFNG* 2109 G/A were investigated as shown in Table 5. When compare PTB patients with LTBI controls (p = 0.004) but not with healthy controls without MTB infection (p = 0.433), the combination of genotypes associated with increased risk of PTB (*TLR9* 1174 G/G, *IFNG* 2109 A/A) was found to contribute to disease development.

**Table 5:** **Combined effect of *TLR9 *** **and *IFNG *** **SNPs on the development of pulmonary tuberculosis**

***TLR9 *** **1174 A/G**	***IFNG *** **2109G/A**	**PTB patients, n (%)**	**LTBI controls n (%)**	Adjusted OR* (95% CI) p value	**Adjusted OR**^**&**^**(95% CI) p value**	**PTB patients, n (%)**	**Healthy controls without MTB infection n (%)**	Adjusted OR* (95% CI) p value	**Adjusted OR**^**&**^**(95% CI)p value**
AA/AG	GG/GA	97 (48.5)	122 (61.9)	Ref.	Ref.	97 (48.5)	103 (52.6)	Ref.	Ref.
AA/AG	AA	62 (31.0)	51 (25.9)	1.54 (0.97-2.43) p = 0.065	**1.97 (1.12-3.47) p = 0.019**	62 (31.0)	60 (30.6)	1.10 (0.70-1.73) p = 0.683	1.02 (0.59-1.77) p = 0.941
G/G	GG/GA	29 (14.5)	18 (9.1)	**2.04 (1.07-3.91) p = 0.031**	2.17 (0.96-4.91) p = 0.062	29 (14.5)	20 (10.2)	1.55 (0.82-2.94) p = 0.178	1.41 (0.65-3.08) p = 0.399
G/G	A/A	12 (6.0)	6 (3.1)	2.53 (0.92-7.00) p = 0.073	**4.31 (0.21-15.31) p = 0.024**	12 (6.0)	13 (6.6)	0.98 (0.43-2.25) p = 0.965	0.95 (0.34-2.62) p = 0.918
p for trend				**0.004**	**0.002**			0.433	0.697

*Abbreviations*: CI confidence interval, LTBI latent M. tuberculosis infection, MTB *Mycobacterium tuberculosis*, OR odds ratio, PTB pulmonary tuberculosis, SNP single nucleotide polymorphism.

*Partly adjusted for age and gender.

^&^Fully adjusted for age, gender, education level, income, body mass index, number of BCG vaccination scars and smoking.

## Discussion

To our knowledge, this is the first large-scale molecular epidemiological study from China that evaluated host genetic susceptibility to PTB development from LTBI. A total of 44 selected SNPs in 32 genes were examined for PTB patients, LTBI controls and healthy controls without MTB infection, respectively. When comparing PTB patients with LTBI controls but not healthy controls without MTB infection, significant associations were observed for *TLR9* 1174 A/G, *TLR9* 1635 A/G and *IFNG* 2109 G/A both individually and in a combined effect model.

It is becoming clear that innate immunity plays an important role in the host defense against MTB infection, and the first step in this process is recognition of MTB [19,20]. Several classes of pattern recognition receptors (PPRs) are involved in the recognition of MTB by cells of the innate immune system. Toll-like receptors (TLRs) are family members of PRRs expressed on macrophages and other leukocytes [21]. These antigen-presenting cells recognize and signal in response to microbial ligands that are bound by the TLRs. As a result, the innate immune system is triggered to produce specific cytokines to contain or eliminate the microbial infection. Among the TLR family, TLR1, TLR2, TLR4, TLR8, and TLR9 and their adaptor molecule MyD88 play the most prominent roles in the initiation of the immune response against MTB [22]. Therefore, as a result, genetic variants of these TLRs have become biologically plausible candidate markers related to host susceptibility. Recently, significant associations between TLR9 polymorphisms and TB disease have been observed in Indonesian [23], Caucasians and Africans [24]. However, such an association was not observed in a Colombian population [25]. Our results suggested an association between TLR9 genetic polymorphisms and the development of PTB from latent infection in Chinese population. TLR9 recognizes unmethylated CpG motifs in bacterial DNA [26]. It is worth noting that it has already been demonstrated that TLR9 recognition modulates the immune response to MTB and cooperates with TLR2 in mediating optimal resistance to this bacterium. TLR2/9 knocks out mice displayed markedly enhanced susceptibility to infection in association with combined defects in pro-inflammatory cytokine production in vitro, IFN-γ recall responses ex vivo, and altered pulmonary pathology [26].

IFN-γ is one of the most widely studied candidate gene potentially associated with TB susceptibility. Positive associations with PTB susceptibility have been observed for multiple SNPs as assessed by individual studies or by means of meta-analysis in different populations including +874 T/A [27,28], 2109 A/G [27], -1616 G/A and 3234 T/C [29]. INF-γ is produced predominantly by natural killer and natural killer T cells as part of the innate immune response, and by CD4 and CD8 T cells once antigen-specific immunity develop [30]. Convincing evidence for its importance in controlling MTB infection has been found by both experimental and clinical studies. Subjects defective in the genes for IFN-γ or IFN-γ receptor have been shown to be prone for MTB infections [31]. In addition, the increased production of IFN-γ post-infection is indicative of the risk of developing active TB, and local and systemic IFN-γ levels associate with the severity of the disease [32,33]. Therefore, these findings suggested important roles of INF-γ both in infection acquisition and during disease development. Consistently, our results indicate an increased risk of PTB development from LTBI for 2109 GG genotype carriage. It has been suggested that *IFNG* 2109 G/A polymorphism influences transcription levels of IFN-γ but the underlying mechanism is still unclear [34]. The interaction between SNPs of *TLR9* and *IFNG* further supports a potential role of TLR9/IFN-γ pathway in active PTB development. Previous studies provided intriguing information about TLR9 SNPs and Malaria severity [35,36], the association of TLR9 polymorphisms with altered IFN-γ levels has been suggested to be potential explanation to TLR9 mediated pathogenesis [37]. However, the association between TLR9/IFN-γ pathway and TB disease severity has not been studied. For the first time, out findings provide a clue for exploring the effects of TLR9/IFN-γ pathway on the development of TB beyond controlling of infection acquisition.

It has been discussed that the selection of controls might partly explain the extensively inconsistency across the genetic association studies on TB susceptibility [10,11]. Recent studies have suggested some genes may actually be related to MTB infection but not TB disease development [38,39], while some other studies have suggested some genes may differentiate between LTBI and active TB disease [40,41]. Such information is important in completely understanding the role of these genes in disease pathogenesis and progression. If controls are latently infected, and there is an association seen between a gene and TB, that suggests the gene influences progression from LTBI to TB. However, if controls are uninfected, the stage specific effect of the genetic markers on infection acquisition or disease development (from latent infection to clinical disease) could not be clarified [10]. In our study, significant associations were observed for PTB as compared with LTBI controls but not healthy controls without MTB infection, which suggested a positive role of the significant SNPs in developing active disease. To further investigate the potential influence of the selection of controls, we analyzed the associations of *TLR9/IFNG* SNPs with PTB by combining LTBI controls and healthy controls without MTB infection together. The results showed that no statistically significant relation was found (please refer to Additional file 4). Therefore, only employing healthy controls with unknown status of MTB infection or only negatives to infection might cover the actually existing associations between SNPs and TB disease. However, it is difficult to truly model the two phenotypes (infection vs disease) with a case–control study design. Given only 10% of people with LTBI will progress to active TB, the vast majority of healthy controls would never progress to active disease. So, the difference between LTBI and healthy controls is minimal. It is necessary to perform further functional studies and prospective studies to verify the role of TLR9/IFN-γ pathway during the natural history of TB development.

In the interpretation of our results, some limitations have to be considered. First, the definition of latent infection based on tuberculin skin test (TST) could not be claimed to be perfect especially among the subjects with BCG vaccination despite our study participants were adults. As reported that on average 30%-35% will have BCG-related positive TST results even after an interval of more than 10 to 15 years [42]. Therefore, TST ≥ 15 mm induration, which should not be attributed to BCG vaccination [43], was used to define latent infection in our present study to minimize potential bias caused by misclassification due to BCG vaccination. In addition, TST is more suitable for epidemiological studies in recourse limited areas as compared to Interferon-Gamma Release Assays (IGRA) which are less influenced by prior BCG vaccination but need more advanced test settings. Second, considering the very low prevalence of HIV among TB patients in China (~ 0.9%) based on our previous systematic review [44], HIV status in the study participants was not considered. Also, there is a difficulty to get inform consent for HIV tests in general population in China due to stigma associated with testing [45]. Third, selected SNPs were tested in this study, potential selection bias caused by such candidate genes approach could not be excluded. In addition, such method was regarded not as comprehensive and unbiased as Genome-wide association studies (GWAS). However, rare GWAS data is available for TB by far. Candidate genes approach is still cost-effective and useful for association analysis especially on polymorphisms with low allele frequencies [46]. Fourth, after correcting for multiple testing with the Bonferroni test as 44 SNPs were tested in the present study, none of the association observed for *TLR9* 1174 A/G, *TLR9* 1635 A/G and *IFNG* 2109 G/A remains statistically significant (p > 0.05/44). Such correction might not be needed since our study mainly addressed moderately well-characterized SNPs with previous suggestions of association with PTB. However, considering this study was conducted in a different population and the strength of the previous data is modest, a possibility of false-positive results in our study could not be completely excluded. Fifth, as the controls were selected from participants of the general health examination, although from the same city, risks of exposure to MTB might be different between the two groups of controls and between PTB patients and controls. Therefore, potential bias caused by selection of controls should be kept in mind when interpreting our results. Further and larger studies, preferably large collaborative studies conducted in different populations, are needed to verify our findings.

## Conclusions

In conclusion, potential associations were observed between TLR9 and IFN-γ genetic polymorphisms and PTB development from LTBI in a Chinese population. These epidemiological findings provided clue for further studies to explore the potential role of TLR9/IFN-γ pathway in human immune responses to MTB infection and active disease development.

## Abbreviations

BCG: Bacille Calmette Guerin; GWAS: Genome-wide association studies; TB: Tuberculosis; HWE: Hardy-Weinberg Equilibrium; IGRA: Interferon-gamma release assays; LTBI: Latent M. tuberculosis infection; MTB: Mycobacterium tuberculosis; PTB: Pulmonary tuberculosis; SNPs: Single nucleotide polymorphisms; TLRs: Toll-like receptors; TST: Tuberculin skin test.

## Competing interests

The authors declare that they have no competing interests.

## Authors’ contributions

LG and LJM conceived and designed the study; YY and XWL carried out the study participants’ enrollment, samples collection and tests; JML, FZ, CG, LG, QH and MFL participated in the questionnaire collection and data management quality control; WC, XJS and QH participated in study coordination; YY, LG and JML performed the statistical analysis and wrote the manuscript; All authors read and approved the final manuscript.

## Pre-publication history

The pre-publication history for this paper can be accessed here:

http://www.biomedcentral.com/1471-2334/13/511/prepub

## Supplementary Material

Additional file 1 The questionnaire used in the study. Click here for file

Additional file 2 List of selected single nucleotide polymorphisms. Click here for file

Additional file 3 The association of selected SNPs with the development of pulmonary tuberculosis. Click here for file

Additional file 4 The association of TLR9 and IFN-γ SNPs with TB (PTB patients v.s. combined controls). Click here for file
