# Supplementary material for: Potential association of pulmonary tuberculosis with genetic polymorphisms of toll-like receptor 9 and interferon-gamma in a Chinese population
Source: BMC Infect Dis. 2013 Oct 31;13:511. doi: 10.1186/1471-2334-13-511 (PMC3819710; doi:10.1186/1471-2334-13-511)
Supplement: Additional file 1 — The questionnaire used in the study. [file 1471-2334-13-511-S1.doc]

**Questionnaire on pulmonary tuberculosis related risk factors**

Interviewer: Date :└─┴─┴─┴─┘(Year)└─┴─┘(Month) └─┴─┘(Day)

Group:[ ] 1）PTB cases； 2）LTBI controls；3）healthy controls without MTB infection

Source:[ ] ①Tuberculosis clinic; ②Other clinics;③Hospitalization tuberculosis patient; ④Other hospitalization patient; ⑤Close contacts; ⑥Physical examination; ⑦hospital staffs and relatives; ⑧Others

Medical record No.: （only for PTB patients）

| **Section 1. General Information** | |
| --- | --- |
| 1 | Gender : [ ] ①male; ②female |
| 2 | Date of Birth : **(**Year) **(**Month); or Age[ ] years |
| 3 | Ethnicity : [ ] ①Han; ②Hui; ③Other |
| 4 | Profession : [ ] ①Administrative staff/Civil servants; ②Technician; ③Worker; ④Teacher; ⑤Student; ⑥Doctor/Nurse; ⑦Office worker; ⑧Self-employed entrepreneurs; ⑨Driver; ⑩Peasant laborer; Farmer; Retiree; Unemployment; Housewife; Other |
| 5 | What is air pollution level of your working/studying environment:[ ]  ①Serious; ②Medium; ③Slight |
| 6 | Education : [ ] ①None; ②Primary school; ③Junior high school; ④Senior high school; ⑤College; ⑥Graduate or above |
| 7 | Marriage status: [ ] ①Married; ②Unmarried; ③Divorced; ④Widowed; ⑤Not clear/Refusal |
| 8 | Your registered permanent residence: [ ] ①This city; ②This province; ③Other province |
| 9 | Up to present, you have lived in this city for [ ] ①6-12 months; ②12-24 months; ③≥24months |
| 10 | Which area do you live :[ ] ①Urban; ②Suburb; ③Village/town; ④Country side |
| 11 | Do you live with someone?:[ ] ①Alone; ②Live with relatives; ③Dormitory |
| 12 | Your living area is (not include yard) [ ]m^2^, and How many people live in your place (include yourself)?[ ] |
| 13 | Which medical insurance do you have? [ ]（You can choose more than one option） ①Basic Medical Insurance; ②Medical services at state expense; ③Urban Employee Basic Medical Insurance; ④New rural cooperative medical care system; ⑤Commercial Medical Insurance; ⑥None |
| 14 | In 2009, your average income per month is [ ] ①None; ②＜500 RMB; ③≥500 RMB and <1000 RMB; ④≥1000 RMB and <2000 RMB; ⑤≥2000 RMB and <3000 RMB; ⑥≥3000 RMB;⑦Not clear |
| 15 | In 2009, your family average income per month is [ ] ①None; ②＜300 RMB; ③≥300 RMB and <500 RMB; ④≥500 RMB and <1000 RMB; ⑤≥1000 RMB and <2000 RMB; ⑥≥2000 RMB;⑦Not clear |
| 16 | Have your family been listed as poverty family? [ ] ①Yes; ②No |
| **Section 2. Life Style** | |
| 17 | Do you smoke? [ ] ①No, I don’t; ②Yes I was, but I have quit ; ③Yes, I smoke  Please jump to 22 if the answer is “No” |
| 18 | What is your age when the first time you smoke? [ ] Please fill in “999” if it is not clear |
| 19 | The average number of cigarettes you smoke per day now or before you quit is [ ] ①≤1; ②2-10; ③11-20; ④≥20 |
| 20 | Do/did you drink?（Beer、White spirit、Wine）:[ ] ①Yes; ②No Please jump to 24 if the answer is “No” |
| 21 | What is your age when the first time you drink? [ ]。 Please fill in “999” if it is not clear |
| 22 | In the past year, how often did you drink? [ ]  ①At least once per day; ②4-6 times per week; ③2-3 times per week; ④1-4 times per month; ⑤Less than once per month |
| 23 | In the past year, how much did you drink per week? Please fill in “0” if do not drink certain kind of alcohol  Beer[ ]bottle（600ml per bottle）; White spirit [ ]liang（50ml per Liang）; Wine[ ]ml |
| 24 | How often do you eat fruits? [ ] ①Every day; ②4-6 day per week; ③2-3 day per week; ④Less than one day per week |
| 25 | How often do you eat vegetables? [ ] ①Every day; ②4-6 day per week; ③2-3 day per week; ④Less than one day per week |
| 26 | How often do you eat eggs? [ ] ①Every day; ②4-6 day per week; ③2-3 day per week; ④Less than one day per week |
| 27 | How often do you eat meat? [ ] ①Every day; ②4-6 day per week; ③2-3 day per week; ④Less than one day per week |
| 28 | Your average sleep time is [ ] ①＜5 hours; ②5-7 hours; ③7-9 hours; ④＞9 hours |
| 29 | Do you ventilate your room every day? [ ] ①Yes; ②No |
| **Section 3. Health Status** | |
| 30 | Height[ ]cm，Weight[ ]Kg，Waist [ ]cm |
| 31 | Blood pressure:[ / ]mmHg |
| 32 | Number of scars by BCG vaccination [ ] ①None; ②One; ③Two; ④Three or more |
| 33 | Have you been diagnosed with tuberculosis? [ ] ①Yes; ②No |
| 34 | Have your lineal relatives been diagnosed with tuberculosis? [ ] ①Yes; ②No |
| 35 | Did you have close contact with tuberculosis patient? [ ] ①Yes; ②No; ③Not clear |
| 36 | Have you been diagnosed with lung cancer?[ ] ①Yes; ②No |
| 37 | Have your lineal relatives (Parents, Son or daughter, Grandparents, Maternal grandparents) been diagnosed with lung cancer? [ ] ①Yes; ②No |
| 38 | Have you been diagnosed with other lung disease(s)?[ ] ①Yes; ②No If the answer is “Yes” The disease(s) is(are) |
| 39 | Have you been diagnosed with diabetes (type I/II) ?[ ] ①Yes; ②No Jump to 48 if the answer is “No” |
| 40 | Have you been receiving treatment for diabetes? [ ] ①Yes; ②No |
| 41 | Have you been diagnosed with hepatitis?[ ] You can choose more than one option ①No; ②Hepatitis A; ③Hepatitis B; ④Hepatitis C; ⑤Other |
| 42 | Have you been diagnosed with the following immune system diseases? [ ] You can choose more than one option ①No; ②Hyperthyroidism; ③Type I diabetes; ④Myasthenia gravis; ⑤Systemic lupus erythematosus; ⑥rheumatoid arthritis; ⑦Ankylosing Spondylitis; ⑧AIDS;⑨Other |
| 43 | Have you accepted transplant operation?[ ] ①Yes; ②No |
| 44 | Have you been receiving treatment of immunosuppressive agent? [ ] ①Yes; ②No |

**Assessor signature:**
