# Supplementary material for: Potential association of pulmonary tuberculosis with genetic polymorphisms of toll-like receptor 9 and interferon-gamma in a Chinese population
Source: BMC Infect Dis. 2013 Oct 31;13:511. doi: 10.1186/1471-2334-13-511 (PMC3819710; doi:10.1186/1471-2334-13-511)
Supplement: Additional file 2 — List of selected single nucleotide polymorphisms. [file 1471-2334-13-511-S2.pdf]

Additional file 1. List of selected single nucleotide polymorphisms

Gene	SNP	dbSNP ID	References
IL1RN	9589 A/T	rs454078	2, 56
IL1A	-889 C/T	rs1800587	3, 15, 31
IL1B	-511 C/T	rs16944	2, 3, 14, 31
IL2	+3896 G/A	rs2069778	17, 18, 31
	-330 T/G	rs2069762	
IL4	-589 C/T	rs2243250	17, 19, 31
IL8	-251T/A	rs4073	21-23
IL10	-819 T/C	rs1800871	31, 2, 3,12,13
	-1082 A/G	rs1800896	
IL18	-137 G/C	rs187238	28-30
	-607 A/C	rs1946518	
TLR-1	-7202 A/G	rs5743551	55
TLR-2	2258 G/A (Arg753Gln)	rs5743708	1, 3, 10
	597 C/T	rs3804099	
TLR4	896 A/G (Asp299Gly)	rs5030729	33, 40, 41
	1196 C/T	rs4986791	
TLR5	1174 C/T	rs5744168	60
TLR8	1 A/G	rs3764880	52
TLR9	1635A/G	rs352140	53, 54
	1174 A/G (P545P)	rs352139	
TLR7	Gln11Leu	rs179008	58, 59
TIRAP	539 C/T	rs8177374	16, 33, 34
SLC11A1	3' UTR (1729+55del4)	rs17235416	3-7
	D543N (Asp543Asn)	rs17235409	
P2X7	1513 A/C	rs3751143	2,3, 9, 8,
	-762 T/C	rs2393799	
CD4	868 C/T	rs28919570	57
CD14	159 C/T	rs2569190	37-39
TNFα	-308 G/A	rs1800629	2, 20, 31, 36
NOD2/CARD15	2104 C/T (Arg702Trp)	rs2066844	42-44
	2722 G/C (Gly908Arg)	rs2066845	
IFNγ	2109 G/A	rs1861494	1
CCR2	190 A/G (V64L)	rs1799864	50
CCR5	59029 A/G	rs1799987	51
MCP-1/CCL2	2518 A/G	rs1024611	3,11,24,25
RANTES/CCL5	-403 G/A	rs2107538	26, 32
	-28 C/G	rs2280788	
CXCR1（IL8RA）	827 G>C (S276T)	rs2234671	48, 49
CXCR2（IL8RB）	768 C/T	rs11574750	
	1208 C/T	rs1126579	
MBL	-221G/C	rs7096206	27, 46, 47
TGFB1	-509 C/T	rs1800469	2, 31, 35
IFKBIA	-881A/G	rs3138053	45
	-826 C/T	rs2233406	

Reference:

1. Leandro AC, Rocha MA, Cardoso CS, Bonecini-Almeida MG. Genetic polymorphisms in vitamin D receptor, vitamin D-binding protein, Toll-like receptor 2, nitric oxide synthase 2, and interferon-gamma genes and its association with susceptibility to tuberculosis. Braz J Med Biol Res. 2009 Apr;42(4):312-22.
2. Taype CA, Shamsuzzaman S, Accinelli RA, Espinoza JR, Shaw MA. Genetic susceptibility to different clinical forms of tuberculosis in the Peruvian population. Infect Genet Evol. 2010 May;10(4):495-504.
3. Motsinger-Reif AA, Antas PR, Oki NO, Levy S, Holland SM, Sterling TR. Polymorphisms in IL-1beta, vitamin D receptor Fok1, and Toll-like receptor 2 are associated with extrapulmonary tuberculosis. BMC Med Genet. 2010 Mar 2;11:37.
4. Li HT, Zhang TT, Zhou YQ, Huang QH, Huang J: SLC11A1 (formerly NRAMP1) gene polymorphisms and tuberculosis susceptibility: a metaanalysis. Int J Tuberc Lung Dis 2006, 10:3-12.
5. Ryu S, Park YK, Bai GH, Kim SJ, Park SN, Kang S: 3'UTR polymorphisms in the NRAMP1 gene are associated with susceptibility to tuberculosis in Koreans. Int J Tuberc Lung Dis 2000, 4:577-580.
6. Liu W, Cao WC, Zhang CY, Tian L, Wu XM, Habbema JD, Zhao QM, Zhang PH, Xin ZT, Li CZ, Yang H: VDR and NRAMP1 gene polymorphisms in susceptibility to pulmonary tuberculosis among the Chinese Han population: a case-control study. Int J Tuberc Lung Dis 2004, 8:428-434.
7. Bellamy R, Ruwende C, Corrah T, McAdam KPWJ, Whittle HC, Hill AVS: Variations in the NRAMP1 gene and susceptibility to tuberculosis in West Africans. N Engl J Med 1998, 338:640-644.
8. Li CM, Campbell SJ, Kumararatne DS, Bellamy R, Ruwende C, McAdam KP, Hill AV, Lammas DA: Association of a polymorphism in the P2X7 gene with tuberculosis in a Gambian population. J Infect Dis 2002, 186:1458-1462.
9. Fernando SL, Saunders BM, Sluyter R, Skarratt KK, Goldberg H, Marks GB, Wiley JS, Britton WJ: A polymorphism in the P2X7 gene increases susceptibility to extrapulmonary tuberculosis. Am J Respir Crit Care Med 2007, 175:360-366.
10. Ogus AC, Yoldas B, Ozdemir T, Uguz A, Olcen S, Keser I, Coskun M, Cilli A, Yegin O: The Arg753GLn polymorphism of the human toll-like receptor 2 gene in tuberculosis disease. Eur Respir J 2004, 23:219-223.
11. Flores-Villanueva PO, Ruiz-Morales JA, Song CH, Flores LM, Jo EK, Montano M, Barnes PF, Selman M, Granados J: A functional promoter polymorphism in monocyte chemoattractant protein-1 is associated with increased susceptibility to pulmonary tuberculosis. J Exp Med 2005, 202:1649-1658.
12. Delgado JC, Baena A, Thim S, Goldfeld AE: Ethnic-specific genetic associations with pulmonary tuberculosis. J Infect Dis 2002, 186:1463-1468.
13. Oral HB, Budak F, Uzaslan EK, Basturk B, Bekar A, Akalin H, Ege E, Ener B, Goral G: Interleukin-10 (IL-10) gene polymorphism as a potential host susceptibility factor in tuberculosis. Cytokine 2006, 35:143-147.
14. Awomoyi AA, Charurat M, Marchant A, Miller EN, Blackwell JM, McAdam KP, Newport MJ: Polymorphism in IL1B: IL1B-511 association with tuberculosis and decreased lipopolysaccharide-induced IL-1beta in IFNgamma primed ex-vivo whole blood assay. J Endotoxin Res 2005, 11:281-286.
15. Amirzargar AA, Rezaei N, Jabbari H, Danesh AA, Khosravi F, Hajabdolbaghi M, Yalda A, Nikbin B: Cytokine single nucleotide polymorphisms in Iranian patients with pulmonary tuberculosis. Eur Cytokine Netw 2006, 17:84-89.
16. Dissanayeke SR, Levin S, Pienaar S, Wood K, Eley B, Beatty D, Henderson H, Anderson S, Levin M. Polymorphic variation in TIRAP is not associated with susceptibility to childhood TB but may determine susceptibility to TBM in some ethnic groups. PLoS One. 2009 Aug 20;4(8):e6698.
17. Yim JJ, Selvaraj P. Genetic susceptibility in tuberculosis. Respirology. 2010 Feb;15(2):241-56.
18. Selvaraj P, Alagarasu K, Harishankar M et al. Cytokine gene polymorphisms and cytokine levels in pulmonary tuberculosis. Cytokine 2008; 43: 26–33.\
19. Amirzargar AA, Rezaei N, Jabbari H et al. Cytokine single nucleotide polymorphisms in Iranian patients with pulmonary tuberculosis. Eur. Cytokine Netw. 2006; 17: 84–9.
20. Selvaraj P, Sriram U, Mathan Kurian S et al. Tumour necrosis factor alpha (-238 and -308) and beta gene polymorphisms in pulmonary tuberculosis: haplotype analysis with HLA-A, B and DR genes. Tuberculosis (Edinb) 2001; 81: 335–41.
21. Ma X, Reich RA, Wright JA et al. Association between interleukin-8 gene alleles and human susceptibility to tuberculosis disease. J. Infect. Dis. 2003; 188: 349–55.
22. Selvaraj P, Prabhuanand S, Jawahar MS et al. Promoter polymorphism of IL-8 gene and IL-8 production in pulmonary tuberculosis. Curr. Sci. 2006; 90: 952–4.
23. Cooke GS, Campbell SJ, Fielding K et al. Interleukin-8 polymorphism is not associated with pulmonary tuberculosis in the gambia. J. Infect. Dis. 2004; 189: 1545–6.
24. Jamieson SE,Miller EN, Black GF et al. Evidence for a cluster of genes on chromosome 17q11-q21 controlling susceptibility to tuberculosis and leprosy in Brazilians. Genes. Immun. 2004; 5: 46–57.
25. Flores-Villanueva PO, Ruiz-Morales JA, Song CH et al. A functional promoter polymorphism in monocyte chemoattractant protein-1 is associated with increased susceptibility to pulmonary tuberculosis. J. Exp.Med. 2005; 202: 1649–58.
26. Chu SF, Tam CM, Wong HS et al. Association between CCL5 functional polymorphisms and tuberculosis in Hong Kong Chinese. Genes Immun. 2007; 8: 475–9.
27. Singla N, Gupta D, Joshi A, Batra N, Singh J, Birbian N. Association of mannose-binding lectin gene polymorphism with tuberculosis susceptibility and sputum conversion time. Int J Immunogenet. 2012 Feb;39(1):10-4.
28. Lee SH, Choi IH, Jeon YK, Park SJ, Lee HK, Lee YM, Chang CL, Kim YS, Lee MK, Park SK. Association between the

interleukin-18 promoter polymorphism and pulmonary tuberculosis in a Korean population. Int J Tuberc Lung Dis. 2011 Sep;15(9):1246-51, i.

29. Han M, Yue J, Lian YY, Zhao YL, Wang HX, Liu LR. Relationship between single nucleotide polymorphism of interleukin-18 and susceptibility to pulmonary tuberculosis in the Chinese Han population. Microbiol Immunol. 2011 Jun;55(6):388-93.
30. Taheri M, Hashemi-Shahri SM, Hamzehnejadi M, Naderi M, Moazeni-Roodi A, Bahari G, Hashemi M. Lack of association between interleukin-18 -607 C/A gene polymorphism and pulmonary tuberculosis in Zahedan, Southeast Iran. Prague Med Rep. 2012;113(1):16-22.
31. Amirzargar AA, Rezaei N, Jabbari H, Danesh AA, Khosravi F, Hajabdolbaghi M, Yalda A, Nikbin B. Cytokine single nucleotide polymorphisms in Iranian patients with pulmonary tuberculosis. Eur Cytokine Netw. 2006 Jun;17(2):84-9.
32. Selvaraj P, Alagarasu K, Singh B, Afsal K. CCL5 (RANTES) gene polymorphisms in pulmonary tuberculosis patients of south India. Int J Immunogenet. 2011 Oct;38(5):397-402.
33. Selvaraj P, Harishankar M, Singh B, Jawahar MS, Banurekha VV. Toll-like receptor and TIRAP gene polymorphisms in pulmonary tuberculosis patients of South India. Tuberculosis (Edinb). 2010 Sep;90(5):306-10.
34. Zhang YX, Xue Y, Liu JY, Zhao MY, Li FJ, Zhou JM, Wang HJ, Li JC. Association of TIRAP (MAL) gene polymorhisms with susceptibility to tuberculosis in a Chinese population. Genet Mol Res. 2011 Jan 4;10(1):7-15.
35. Mak JC, Leung HC, Sham AS, Mok TY, Poon YN, Ling SO, Wong KC, Chan-Yeung M. Genetic polymorphisms and plasma levels of transforming growth factor-beta(1) in Chinese patients with tuberculosis in Hong Kong. Cytokine. 2007 Dec;40(3):177-82.
36. Fan HM, Wang Z, Feng FM, Zhang KL, Yuan JX, Sui H, Qiu HY, Liu LH, Deng XJ, Ren JX. Association of TNF-alpha-238G/A and 308 G/A gene polymorphisms with pulmonary tuberculosis among patients with coal worker's pneumoconiosis. Biomed Environ Sci. 2010 Apr;23(2):137-45.
37. Kang YA, Lee HW, Kim YW, Han SK, Shim YS, Yim JJ. Association between the -159C/T CD14 gene polymorphism and tuberculosis in a Korean population. FEMS Immunol Med Microbiol. 2009 Dec;57(3):229-35.
38. Rosas-Taraco AG, Revol A, Salinas-Carmona MC, Rendon A, Caballero-Olin G, Arce-Mendoza AY. CD14 C(-159)T polymorphism is a risk factor for development of pulmonary tuberculosis. J Infect Dis. 2007 Dec 1;196(11):1698-706.
39. Pacheco E, Fonseca C, Montes C, Zabaleta J, García LF, Arias MA. CD14 gene promoter polymorphism in different clinical forms of tuberculosis. FEMS Immunol Med Microbiol. 2004 Apr 9;40(3):207-13.
40. Pulido I, Leal M, Genebat M, Pacheco YM, Sáez ME, Soriano-Sarabia N. The TLR4 ASP299GLY polymorphism is a risk factor for active tuberculosis in Caucasian HIV-infected patients. Curr HIV Res. 2010 Apr;8(3):253-8.
41. Ferwerda B, Kibiki GS, Netea MG, Dolmans WM, van der Ven AJ. The toll-like receptor 4 Asp299Gly variant and tuberculosis susceptibility in HIV-infected patients in Tanzania. AIDS. 2007 Jun 19;21(10):1375-7.
42. Stockton JC, Howson JM, Awomoyi AA, McAdam KP, Blackwell JM, Newport MJ. Polymorphism in NOD2, Crohn's disease, and susceptibility to pulmonary tuberculosis. FEMS Immunol Med Microbiol. 2004 Jun 1;41(2):157-60.
43. Möller M, Nebel A, Kwiatkowski R, van Helden PD, Hoal EG, Schreiber S. Host susceptibility to tuberculosis: CARD15 polymorphisms in a South African population. Mol Cell Probes. 2007 Apr;21(2):148-51.
44. Austin CM, Ma X, Graviss EA. Common nonsynonymous polymorphisms in the NOD2 gene are associated with resistance or susceptibility to tuberculosis disease in African Americans. J Infect Dis. 2008 Jun 15;197(12):1713-6.
45. Sun XF, Zhang H. NFKB and NFKBI polymorphisms in relation to susceptibility of tumour and other diseases. Histol Histopathol. 2007 Dec;22(12):1387-98.
46. Denholm JT, McBryde ES, Eisen DP. Mannose-binding lectin and susceptibility to tuberculosis: a meta-analysis. Clin Exp Immunol. 2010 Oct;162(1):84-90.
47. Liu W, Zhang F, Xin ZT, Zhao QM, Wu XM, Zhang PH, de Vlas S, Richardus JH, Habbema JD, Yang H, Cao WC. Sequence variations in the MBL gene and their relationship to pulmonary tuberculosis in the Chinese Han population. Int J Tuberc Lung Dis. 2006 Oct;10(10):1098-103.
48. Kormann MS, Hector A, Marcos V, Mays LE, Kappler M, Illig T, Klopp N, Zeilinger S, Carevic M, Rieber N, Eickmeier O, Zielen S, Gaggar A, Moepps B, Griese M, Hartl D. CXCR1 and CXCR2 haplotypes synergistically modulate cystic fibrosis lung disease. Eur Respir J. 2012 Jun;39(6):1385-90.
49. Stemmler S, Arinir U, Klein W, Rohde G, Hoffjan S, Wirkus N, Reinitz-Rademacher K, Bufe A, Schultze-Werninghaus G, Epplen JT. Association of interleukin-8 receptor alpha polymorphisms with chronic obstructive pulmonary disease and asthma. Genes Immun. 2005 May;6(3):225-30.
50. Alagarasu K, Selvaraj P, Swaminathan S, Raghavan S, Narendran G, Narayanan PR. CCR2, MCP-1, SDF-1a & DC-SIGN gene polymorphisms in HIV-1 infected patients with & without tuberculosis. Indian J Med Res. 2009 Oct;130(4):444-50.
51. Mamtani M, Mummidi S, Ramsuran V, Pham MH, Maldonado R, Begum K, Valera MS, Sanchez R, Castiblanco J, Kulkarni H, Ndung'u T, He W, Anaya JM, Ahuja SK. Influence of variations in CCL3L1 and CCR5 on tuberculosis in a northwestern Colombian population. J Infect Dis. 2011 Jun 1;203(11):1590-4.
52. Davila S, Hibberd ML, Hari Dass R, Wong HE, Sahiratmadja E, Bonnard C, Alisjahbana B, Szeszko JS, Balabanova Y, Drobniewski F, van Crevel R, van de Vosse E, Nejentsev S, Ottenhoff TH, Seielstad M. Genetic association and expression studies indicate a role of toll-like receptor 8 in pulmonary tuberculosis. PLoS Genet. 2008 Oct;4(10):e1000218.
53. Kobayashi K, Yuliwulandari R, Yanai H, Naka I, Lien LT, Hang NT, Hijikata M, Keicho N, Tokunaga K. Association of

TLR polymorphisms with development of tuberculosis in Indonesian females. Tissue Antigens. 2012 Mar;79(3):190-7.

54. Kleinnijenhuis J, Oosting M, Joosten LA, Netea MG, Van Crevel R. Innate immune recognition of Mycobacterium tuberculosis. Clin Dev Immunol. 2011;2011:405310.
55. Randhawa AK, Shey MS, Keyser A, Peixoto B, Wells RD, de Kock M, Lerumo L, Hughes J, Hussey G, Hawkridge A, Kaplan G, Hanekom WA, Hawn TR; South African Tuberculosis Vaccine Initiative Team. Association of human TLR1 and TLR6 deficiency with altered immune responses to BCG vaccination in South African infants. PLoS Pathog. 2011 Aug;7(8):e1002174.
56. Awomoyi AA, Charurat M, Marchant A, Miller EN, Blackwell JM, McAdam KP, Newport MJ. Polymorphism in IL1B: IL1B-511 association with tuberculosis and decreased lipopolysaccharide-induced IL-1beta in IFN-gamma primed ex-vivo whole blood assay. J Endotoxin Res. 2005;11(5):281-6.
57. Choi RY, Farquhar C, Juno J, Mbori-Ngacha D, Lohman-Payne B, Vouriot F, Wayne S, Tuff J, Bosire R, John-Stewart G, Fowke K. Infant CD4 C868T polymorphism is associated with increased human immunodeficiency virus (HIV-1) acquisition. Clin Exp Immunol. 2010 Jun;160(3):461-5.
58. Lombardi V, Van Overtvelt L, Horiot S, Moingeon P. Human dendritic cells stimulated via TLR7 and/or TLR8 induce the sequential production of Il-10, IFN-gamma, and IL-17A by naive CD4+ T cells. J Immunol. 2009 Mar 15;182(6):3372-9.
59. Oh DY, Baumann K, Hamouda O, Eckert JK, Neumann K, Kücherer C, Bartmeyer B, Poggensee G, Oh N, Pruss A, Jessen H, Schumann RR. A frequent functional toll-like receptor 7 polymorphism is associated with accelerated HIV-1 disease progression. AIDS. 2009 Jan 28;23(3):297-307.
60. Merx S, Zimmer W, Neumaier M, Ahmad-Nejad P. Characterization and functional investigation of single nucleotide polymorphisms (SNPs) in the human TLR5 gene. Hum Mutat. 2006 Mar;27(3):293.
