# Supplementary material for: Potential association of pulmonary tuberculosis with genetic polymorphisms of toll-like receptor 9 and interferon-gamma in a Chinese population
Source: BMC Infect Dis. 2013 Oct 31;13:511. doi: 10.1186/1471-2334-13-511 (PMC3819710; doi:10.1186/1471-2334-13-511)
Supplement: Additional file 3 — The association of selected SNPs with the development of pulmonary tuberculosis. [file 1471-2334-13-511-S3.pdf]

Additional file 2. The association of selected SNPs with the development of pulmonary tuberculosis (Part 1/4)

Genotype		PTB patients	LTBI controls	Adjusted OR* (95% CI), p value	PTB patients	Healthy controls	Adjusted OR* (95% CI) , p value
IL1RN 9589 A/T (rs454078)	TT	170	169	Ref.	170	173	Ref.
	TA	29	28	1.03 (0.59-1.82), p=0.908	29	23	1.29 (0.71-2.32), p=0.402
	AA	1	0	NA	1	0	NA
	Dominant	AA+TA vs TT		1.07 (0.61-1.88), p=0.819			1.33 (0.74-2.39) , p=0.340
	Recessive	AA vs TA+TT		NA			NA
IL1B -511 C/T (rs16944)	CC	54	63	Ref.	54	59	Ref.
	CT	96	97	1.15 (0.73-1.83) , p=0.541	96	99	1.06 (0.67-1.69) , p=0.807
	TT	50	37	1.58 (0.90-2.77) , p=0.109	50	38	1.44 (0.82-2.52) , p=0.204
	Dominant	TT+CT vs CC		1.45 (0.89-2.34) , p=0.134			1.39 (0.86-2.23) , p=0.179
	Recessive	TT vs CT+CC		1.27 (0.83-1.96) , p=0.117			1.17 (0.75-1.80) , p=0.494
IL2 -330 T/G (rs2069762)	TT	89	90	Ref.	89	87	Ref.
	TG	88	85	1.05 (0.69-1.59) , p=0.831	88	88	0.98 (0.64-1.48) , p=0.913
	GG	23	22	1.06 (0.55-2.03) , p=0.867	23	21	1.07 (0.55-2.09) , p=0.835
	Dominant	GG+TG vs TT		1.05 (0.71-1.56) , p=0.813			1.00 (0.67-1.48) , p=0.982
	Recessive	GG vs TG+TT		1.03 (0.56-1.93) , p=0.916			1.09 (0.58-2.04) , p=0.798
IL4 -589 C/T (rs2243250)	TT	120	106	Ref.	120	105	Ref.
	TC	70	79	0.78 (0.52-1.18) , p=0.246	70	77	0.80 (0.52-1.21) , p=0.282
	CC	10	12	0.73 (0.30-1.77) , p=0.489	10	14	0.63 (0.27-1.47) , p=0.280
	Dominant	CC+TC vs TT		0.78 (0.52-1.16) , p=0.631			0.77 (0.52-1.15) , p=0.197
	Recessive	CC vs TC+TT		0.81 (0.34-1.93) , p=0.212			0.69 (0.30-1.58) , p=0.375
IL8 -251 T/A (rs4073)	AA	74	75	Ref.	74	68	Ref.
	AT	97	93	1.06 (0.69-1.63) , p=0.796	97	99	0.90 (0.58-1.39) , p=0.636
	TT	29	29	1.01 (0.55-1.86) , p=0.966	29	29	0.92 (0.50-1.70) , p=0.788
	Dominant	TT+AT vs AA		1.05 (0.70-1.57) , p=0.823			0.91 (0.60-1.37) , p=0.634
	Recessive	TT vs AT+AA		0.98 (0.56-1.72) , p=0.949			0.98 (0.56-1.71) , p=0.935
IL8RB 1208 C/T (rs1126579)	TT	83	83	Ref.	83	78	Ref.
	TC	85	82	1.04 (0.67-1.59) , p=0.871	85	88	0.91 (0.59-1.40) , p=0.656
	CC	32	32	1.00 (0.56-1.79) , p=0.998	32	30	1.00 (0.55-1.81) , p=0.998
	Dominant	CC+TC vs TT		1.03 (0.69-1.53) , p=0.899			0.93 (0.62-1.39) , p=0.724
	Recessive	CC vs TC+TT		0.98 (0.57-1.68) , p=0.951			1.05 (0.61-1.82) , p=0.850
IL10 -819 T/C (rs1800871)	TT	99	83	Ref.	99	81	Ref.
	TC	80	94	0.71 (0.47-1.08) , p=0.113	80	87	0.75 (0.49-1.15) , p=0.182
	CC	21	20	0.88 (0.45-1.74) , p=0.712	21	28	0.62 (0.33-1.16) , p=0.134
	Dominant	CC+TC vs TT		0.72 (0.50-1.10) , p=0.141			0.71 (0.48-1.06) , p=0.099
	Recessive	CC vs TC+TT		1.04 (0.54-1.99) , p=0.908			0.70 (0.38-1.29) , p=0.256
IL10 -1082 A/G (rs1800896)	AA	176	162	Ref.	176	160	Ref.
	AG	23	35	0.60 (0.34-1.07), p=0.082	23	35	0.60 (0.34-1.05) , p=0.075
	GG	1	0	NA	1	1	0.91 (0.06-14.69) , p=0.945
	Dominant	GG+AG vs AA		0.63 (0.36-1.11) , p=0.108			0.61 (0.35-1.06) , p=0.078
	Recessive	GG vs AG+AA		NA			0.98 (0.06-15.82) , p=0.988

Abbreviation: CI, confidence interval; LTBI, latent M. tuberculosis infection; NA, not available; OR, odds ratio; PTB, pulmonary tuberculosis; SNP, single nucleotide polymorphism; TB, tuberculosis.

* Adjusted for age and gender.

Additional file 2. The association of selected SNPs with the development of pulmonary tuberculosis (Part 2/4)

Genotype		PTB patients	LTBI controls	Adjusted OR* (95% CI), p value	PTB patients	Healthy controls	Adjusted OR* (95% CI) , p value
IL18 -137 G/C (rs187238)	CC	157	147	Ref.	157	152	Ref.
	CG	40	46	0.81 (0.50-1.32) , p=0.399	40	42	0.92 (0.57-1.50) , p=0.745
	GG	3	4	0.70 (0.15-3.20) , p=0.647	3	2	1.45 (0.24-8.82) , p=0.685
	Dominant	GG+CG vs CC		0.80 (0.50-1.28) , p=0.361			0.95 (0.59-1.53) , p=0.821
	Recessive	GG vs CG+CC		0.73 (0.16-3.34) , p=0.689			1.48 (0.24-8.94) , p=0.671
IL18 -607 A/C (rs1946518)	CC	58	50	Ref.	58	49	Ref.
	CA	99	101	0.84 (0.53-1.35) , p=0.479	99	101	0.83 (0.51-1.32) , p=0.427
	AA	43	46	0.80 (0.46-1.41) , p=0.448	43	46	0.79 (0.45-1.39) , p=0.409
	Dominant	AA+CAvs CC		0.83 (0.53-1.30) , p=0.416			0.81 (0.52-1.27) , p=0.365
	Recessive	AA vs CA+CC		0.90 (0.56-1.44) , p=0.656			0.89 (0.55-1.43) , p=0.637
TLR1 -7202 A/G (rs5743551)	GG	90	90	Ref.	90	74	Ref.
	GA	84	91	0.91 (0.60-1.38) , p=0.663	84	103	0.66 (0.43-1.01) , p=0.055
	AA	25	16	1.55 (0.77-3.10) , p=0.216	25	19	1.06 (0.54-2.01) , p=0.850
	Dominant	AA+GA vs GG		1.01 (0.68-1.50) , p=0.969			0.72 (0.48-1.08) , p=0.116
	Recessive	AA vs GA+GG		1.62 (0.83-3.14) , p=0.154			1.33 (0.71-2.51) , p=0.377
TLR2 597 C/T (rs3804099)	TT	97	102	Ref.	97	97	Ref.
	TC	83	73	1.20 (0.79-1.82) , p=0.404	83	81	1.03 (0.68-1.56) , p=0.905
	CC	20	22	0.96 (0.49-1.86) , p=0.895	20	18	1.11 (0.55-2.24) , p=0.764
	Dominant	CC+TC vs TT		1.14 (0.77-1.69) , p=0.514			1.04 (0.70-1.55) , p=0.841
	Recessive	CC vs TC+TT		0.88 (0.47-1.68) , p=0.705			1.10 (0.56-2.15) , p=0.781
TLR5 1174 C/T (rs5744168)	CC	189	189	Ref.	189	188	Ref.
	CT	11	8	1.38 (0.54-3.50) , p=0.504	11	7	1.57 (0.60-4.14) , p=0.361
	TT	0	0	NA	0	0	NA
	Dominant	TT+CT vs CC		1.38 0.54-3.50) , p=0.504			1.57 (0.60-4.14) , p=0.361
	Recessive	TT vs CT+CC		NA			NA
CCR2 190 A/G (rs1799864)	GG	125	125	Ref.	125	120	Ref.
	GA	62	65	0.95 (0.62-1.46) , p=0.829	62	67	0.89 (0.58-1.37) , p=0.590
	AA	13	7	1.87 (0.72-4.87) , p=0.200	13	9	1.39 (0.57-3.37) , p=0.469
	Dominant	AA+GA vs GG		1.04 (0.69-1.57) , p=0.842			0.95 (0.63-1.43) , p=0.797
	Recessive	AA vs GA+GG		1.90 (0.74-4.90) , p=0.184			1.45 (0.60-3.47) , p=0.408
CCR5 59029 A/G (rs1799987)	GG	71	62	Ref.	71	59	Ref.
	GA	97	107	0.79 (0.51-1.23) , p=0.293	97	102	0.79 (0.51-1.23) , p=0.590
	AA	32	28	1.00 (0.54-1.85) , p=0.997	32	35	0.76 (0.42-1.37) , p=0.469
	Dominant	AA+GA vs GG		0.83 (0.55-1.27) , p=0.396			0.78 (0.51-1.19) , p=0.251
	Recessive	AA vs GA+GG		1.15 (0.669-2.00) , p=0.616			0.88 (0.52-1.48) , p=0.623
RANTES -403 G/A (rs2107538)	GG	77	63	Ref.	77	74	Ref.
	GA	96	101	0.78 (0.50-1.20) , p=0.256	96	95	0.97 (0.64-1.49) , p=0.893
	AA	27	33	0.67 (0.36-1.23) , p=0.195	27	27	0.96 (0.52-1.79) , p=0.901
	Dominant	AA+GA vs GG		0.75 (0.50-1.14) , p=0.174			0.97 (0.65-1.46) , p=0.879
	Recessive	AA vs GA+GG		0.78 (0.45-1.35) , p=0.366			0.98 (0.55-1.74) , p=0.938

Abbreviation: CI, confidence interval; LTBI, latent M. tuberculosis infection; NA, not available; OR, odds ratio; PTB, pulmonary tuberculosis; SNP, single nucleotide polymorphism; TB, tuberculosis.

* Adjusted for age and gender.

Additional file 2. The association of selected SNPs with the development of pulmonary tuberculosis (Part 3/4)

Genotype		PTB patients	LTBI controls	Adjusted OR* (95% CI), p value	PTB patients	Healthy controls	Adjusted OR* (95% CI) , p value
MCP1 2518 A/G (rs1024611)	GG	71	64	Ref.	71	75	Ref.
	GA	96	104	0.83 (0.54-1.29) , p=0.408	96	88	1.15 (0.75-1.78) , p=0.524
	AA	33	29	1.02 (0.56-1.88) , p=0.941	33	33	1.06 (0.59-1.89) , p=0.856
	Dominant	AA+GA vs GG		0.87 (0.57-1.33) , p=0.522			1.13 (0.75-1.69) , p=0.571
	Recessive	AA vs GA+GG		1.14 (0.66-1.97) , p=0.627			0.98 (0.57-1.65) , p=0.926
NRAMP1 D543N (rs17235409)	GG	151	151	Ref.	151	139	Ref.
	GA	47	43	1.09 (0.68-1.76) , p=0.710	47	55	0.79 (0.50-1.24) , p=0.295
	AA	2	3	0.67 (0.11-4.06) , p=0.661	2	2	0.93 (0.13-6.74) , p=0.945
	Dominant	AA+GA vs GG		1.07 (0.67-1.70) , p=0.785			0.79 (0.51-1.24) , p=0.302
	Recessive	AA vs GA+GG		0.65 (0.11-3.96) , p=0.643			0.99 (0.14-7.12) , p=0.989
P2X7 1513 A/C (rs3751143)	AA	116	113	Ref.	116	109	Ref.
	AC	76	71	1.04 (0.69-1.58) , p=0.850	76	76	0.94 (0.62-1.42) , p=0.767
	CC	8	13	0.60(0.24-1.50) , p=0.270	8	11	0.68 (0.27-1.77) , p=0.431
	Dominant	CC+ACvs AA		0.97 (0.65-1.45) , p=0.894			0.91 (0.61-1.35) , p=0.632
	Recessive	CC vs AC+AA		0.59 (0.24-1.45) , p=0.247			0.70 (0.28-1.78) , p=0.456
P2X7 -762 T/C (rs2393799)	TT	92	86	Ref.	92	86	Ref.
	TC	81	86	0.87 (0.57-1.33) , p=0.521	81	88	0.85 (0.56-1.30) , p=0.458
	CC	26	25	0.96 (0.52-1.79) , p=0.900	26	42	1.10 (0.58-2.08) , p=0.784
	Dominant	CC+TC vs TT		0.89 (0.60-1.33) , p=0.569			0.90 (0.61-1.34) , p=0.604
	Recessive	CC vs TC+TT		1.03 (0.57-1.85) , p=0.928			1.18 (0.65-2.17) , p=0.586
TNF-α -308 G/A (rs1800629)	GG	169	178	Ref.	169	176	Ref.
	GA	29	18	1.71 (0.91-3.20) , p=0.095	29	19	1.60 (0.86-2.96) , p=0.138
	AA	2	1	2.14 (0.19-23.99) , p=0.573	2	1	2.07 (0.19-23.15) , p=0.553
	Dominant	AA+GA vs GG		1.73 (0.94-3.19) , p=0.080			1.62 (0.89-2.96) , p=0.116
	Recessive	AA vs GA+GG		1.98 (0.18-22.17) , p=0.579			1.96 (0.18-21.88) , p=0.583
CD4 868 C/T (rs28919570)	CC	188	185	Ref.	188	184	Ref.
	CT	11	12	0.90 (0.39-2.10) , p=0.810	11	12	0.90 (0.39-2.09) , p=0.802
	TT	1	0	NA	1	0	NA
	Dominant	TT+CT vs CC		0.98 (0.43-2.25) , p=0.970			0.98 (0.43-2.24) , p=0.962
	Recessive	TT vs CT+CC		NA			NA
CD14 159 C/T (rs2569190)	TT	54	66	Ref.	54	60	Ref.
	TC	103	97	1.28 (0.81-2.01) , p=0.293	103	104	1.08 (0.68-1.70) , p=0.745
	CC	42	34	1.48 (0.83-2.64) , p=0.181	42	32	1.43 (0.80-2.58) , p=0.229
	Dominant	CC+TC vs TT		1.33 (0.87-2.04) , p=0.194			1.16 (0.75-1.80) , p=0.497
	Recessive	CC vs TC+TT		1.27 (0.77-2.11) , p=0.344			1.36 (0.82-2.28) , p=0.231
MBL -221 G/C (rs7096206)	CC	150	150	Ref.	150	141	Ref.
	CG	48	45	1.06 (0.67-1.70) , p=0.785	48	52	0.87 (0.55-1.37), p=0.542
	GG	2	2	1.00 (0.14-7.23) , p=0.999	2	3	0.63 (0.10-3.82) , p=0.612
	Dominant	GG+CG vs CC		1.07 (0.67-1.69) , p=0.790			0.86 (0.55-1.34) , p=0.493
	Recessive	GG vs CG+CC		0.99 (0.14-7.10) , p=0.990			0.65 (0.11-3.94) , p=0.640

Abbreviation: CI, confidence interval; LTBI, latent M. tuberculosis infection; NA, not available; OR, odds ratio; PTB, pulmonary tuberculosis; SNP, single nucleotide polymorphism; TB, tuberculosis.

* Adjusted for age and gender.

Additional file 2. The association of selected SNPs with the development of pulmonary tuberculosis (Part 4/4)

Genotype		PTB patients	LTBI controls	Adjusted OR* (95% CI), p value	PTB patients	Healthy controls	Adjusted OR* (95% CI) , p value
TIRAP 539 C/T (rs8177374)	CC	196	190	Ref.	196	188	Ref.
	CT	4	7	0.55 (0.16-1.92), p=0.352	4	7	0.55 (0.16-1.91) , p=0.348
	TT	0	0	NA	0	0	NA
	Dominant	TT+CT vs CC		0.55 (0.16-1.92) , p=0.352			0.55 (0.16-1.91) , p=0.348
	Recessive	TT vs CT+CC		NA			NA
NFKBIA -881 A/G (rs3138053)	AA	150	148	Ref.	150	153	Ref.
	AG	48	47	1.01 (0.63-1.60) , p=0.975	48	43	1.14 (0.71-1.82) , p=0.591
	GG	2	2	0.99 (0.14-7.10) , p=0.989	2	0	NA
	Dominant	TT+AG vs GG		1.01 (0.64-1.59) , p=0.977			1.19 (0.74-1.89) , p=0.474
	Recessive	TT vs AG+GG		0.98 (0.14-7.06) , p=0.988			NA
NFKBIA -826 C/T (rs2233406)	GG	149	148	Ref.	149	153	Ref.
	GA	48	47	1.01 (0.63-1.60) , p=0.975	48	43	1.14 (0.71-1.82) , p=0.591
	AA	2	2	0.99 (0.14-7.10) , p=0.989	2	0	NA
	Dominant	AA+GA vs GG		1.01 (0.64-1.59) , p=0.977			1.02 (0.68-1.53) , p=0.474
	Recessive	AA vs GA+GG		0.98 (0.14-7.06) , p=0.988			NA

Abbreviation: CI, confidence interval; LTBI, latent M. tuberculosis infection; NA, not available; OR, odds ratio; PTB, pulmonary tuberculosis; SNP, single nucleotide polymorphism; TB, tuberculosis.

* Adjusted for age and gender.
