# Supplementary material for: Potential association of pulmonary tuberculosis with genetic polymorphisms of toll-like receptor 9 and interferon-gamma in a Chinese population
Source: BMC Infect Dis. 2013 Oct 31;13:511. doi: 10.1186/1471-2334-13-511 (PMC3819710; doi:10.1186/1471-2334-13-511)
Supplement: Additional file 4 — The association of TLR9 and IFN-γ SNPs with TB (PTB patients v.s. combined controls). [file 1471-2334-13-511-S4.doc]

**Additional file 4. The association of *TLR9* and *IFN-γ* SNPs with TB (PTB patients v.s. combined controls)**

| **Genotype** | | **PTB patients**  **n (%)** | **Controls with LTBI or without infection**  **n (%)** | **Adjusted OR*  (95% CI)**  **p value** | **Adjusted OR^&^  (95% CI)**  **p value** |
| --- | --- | --- | --- | --- | --- |
| *TLR9*  1174 A/G  (rs352139) | AA | 70 (35.0) | 135 (34.4) | Ref. | Ref. |
|  | AG | 89 (44.5) | 201 (51.2) | 0.85 (0.58-1.25)  p=0.410 | 0.75 (0.48-1.18)  p=0.211 |
|  | GG | 41 (20.5) | 57 (14.5) | 1.64 (0.19-3.01)  p=0.190 | 1.34 (0.73-2.45)  p=0.340 |
|  | Dominant | GG+AG vs AA | | 0.97 (0.68-1.39)  p=0.874 | 0.87 (0.57-1.33)  p=0.528 |
|  | Recessive | GG vs AG+AA | | 1.53 (0.99-2.39)  p=0.063 | 1.58 (0.91-2.72)  p=0.102 |
| *TLR9*  1635 A/G  (rs352140) | GG | 72 (36.0) | 141 (35.9) | Ref. | Ref. |
|  | GA | 88 (44.0) | 197 (50.1) | 0.87 (0.60-1.28)  p=0.483 | 0.80 (0.51-1.26)  p=0.336 |
|  | AA | 40 (20.0) | 55 (14.0) | 1.43 (0.87-2.35)  p=0.160 | 1.36 (0.74-2.48)  p=0.323 |
|  | Dominant | AA+GA vs GG | | 0.99 (0.70-1.42)  p=0.975 | 0.92 (0.60-1.40)  p=0.693 |
|  | Recessive | AA vs GA+GG | | 1.54 (0.98-2.42)  p=0.059 | 1.53 (0.88-2.66)  p=0.130 |
| *IFN-γ*  2109 G/A  (rs1861494) | AA | 74 (37.0) | 130 (33.1) | Ref. | Ref. |
|  | AG | 97 (48.5) | 184 (46.8) | 0.93 (0.64-1.35)  p=0.691 | 0.88 (0.56-1.39) p=0.595 |
|  | GG | 29 (14.5) | 79 (20.1) | 0.64 (0.39-1.08)  p=0.093 | 0.56(0.31-1.04)  p=0.068 |
|  | Dominant | GG+AG vs AA | | 0.67 (0.42-1.07)  p=0.095 | 0.61 (0.35-1.06)  p=0.078 |
|  | Recessive | GG vs AG+AA | | 0.84 (0.60-1.20)  p=0.340 | 0.79 (0.51-1.21)  p=0.270 |

Abbreviation: CI, confidence interval; LTBI, latent M. tuberculosis infection; OR, odds ratio; PTB, pulmonary tuberculosis; SNP, single nucleotide polymorphism; TB, tuberculosis.

* Partly adjusted for age and gender.

^&^Fully adjusted for age, gender, education level, income, body mass index, number of BCG vaccination scars and smoking.
